# Methyl isonicotinate 1-oxide

**DOI:** 10.1107/S1600536810004629

**Published:** 2010-02-10

**Authors:** Hui Li, Wen-Ni Zheng

**Affiliations:** a College of Chemistry and Chemical, Engineering, Southeast University, Nanjing 210096, People’s Republic of China

## Abstract

In the title compound, C_7_H_7_NO_3_, the benzene ring and the methyl ester group are nearly coplanar, forming a dihedral of 3.09 (9)°. The crystal structure is stabilized by inter­molecular C—H⋯O hydrogen bonds, forming layers parallel to (101).

## Related literature

For the application of carboxyl­ate derivatives in microelectronics and as memory storage devices, see: Fu *et al.* (2007 ▶, 2008 ▶); Fu & Xiong (2008 ▶).
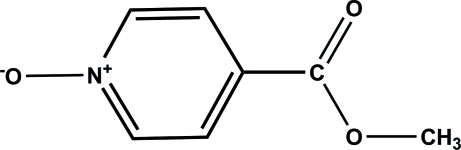


## Experimental

### 

#### Crystal data

C_7_H_7_NO_3_
*M* *_r_* = 153.14
Monoclinic, 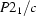

*a* = 7.2429 (14) Å
*b* = 10.347 (2) Å
*c* = 9.898 (2) Å
β = 105.09 (3)°
*V* = 716.2 (3) Å^3^
*Z* = 4
Mo *K*α radiation
μ = 0.11 mm^−1^
*T* = 298 K
0.30 × 0.25 × 0.20 mm

#### Data collection

Rigaku Mercury2 diffractometer
Absorption correction: multi-scan (*CrystalClear*; Rigaku, 2005 ▶) *T*_min_ = 0.96, *T*_max_ = 1.00
7070 measured reflections
1640 independent reflections
972 reflections with *I* > 2σ(*I*)
*R*_int_ = 0.053

#### Refinement

*R*[*F* ^2^ > 2σ(*F* ^2^)] = 0.060
*wR*(*F* ^2^) = 0.180
*S* = 1.02
1640 reflections
100 parameters
H-atom parameters constrained
Δρ_max_ = 0.18 e Å^−3^
Δρ_min_ = −0.16 e Å^−3^

### 

Data collection: *CrystalClear* (Rigaku, 2005 ▶); cell refinement: *CrystalClear*; data reduction: *CrystalClear*; program(s) used to solve structure: *SHELXS97* (Sheldrick, 2008 ▶); program(s) used to refine structure: *SHELXL97* (Sheldrick, 2008 ▶); molecular graphics: *SHELXTL* (Sheldrick, 2008 ▶); software used to prepare material for publication: *SHELXTL*.

## Supplementary Material

Crystal structure: contains datablocks I, New_Global_Publ_Block. DOI: 10.1107/S1600536810004629/rz2415sup1.cif

Structure factors: contains datablocks I. DOI: 10.1107/S1600536810004629/rz2415Isup2.hkl

Additional supplementary materials:  crystallographic information; 3D view; checkCIF report

## Figures and Tables

**Table 1:** Hydrogen-bond geometry (Å, °)

*D*—H⋯*A*	*D*—H	H⋯*A*	*D*⋯*A*	*D*—H⋯*A*
C2—H2*A*⋯O2^i^	0.93	2.44	3.204 (3)	139
C4—H4*A*⋯O3^ii^	0.93	2.42	3.263 (3)	150

Symmetry codes: (i) 
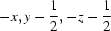
; (ii) 
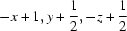
.

**Fig. 1.**
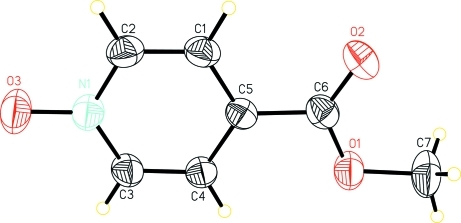
A view of the title compound with the atomic numbering scheme. Displacement ellipsoids were drawn at the 30% probability level.

## supplementary crystallographic information

### Comment

Carboxylate derivatives attracted more attention as pharmaceutical and phase transition dielectric materials for their application in micro-electronics and as memory storage devices (Fu *et al.*, 2007; Fu & Xiong 2008; Fu *et al.*, 2008). With the purpose of obtaining phase transition crystals of carboxylate compounds, the interaction of methyl isonicotinate with hydrogen peroxide has been studied and we have elaborated a series of new materials including these organic molecules. In this paper, we describe the crystal structure of the title compound, Methyl isonicotinate 1-oxide.

In the title compound (Fig. 1), the benzene ring and the methyl ester group are nearly coplanar, the dihedral angle they form being 3.09 (9)°). The N1—O3 bond length of the nitrile group (1.292 (2)Å) is within the normal range. The crystal structure is stabilized by intermolecular C—H···O hydrogen bonds (Table 1) linking the molecules to form layers parallel to  the (101) plane.

### Experimental

Methyl isonicotinate 1-oxide (3 mmol, 0.46 g) was dissolved in methanol. The solvent was slowly evaporated in air affording colourless block-shaped crystals of the title compound suitable for X-ray analysis. Permittivity measurements show that there is no phase transition within the temperature range (from 100 K to 400 K), and the permittivity is 6.5 at 1 MHz at room temperature.

### Refinement

All H atoms attached to C atoms were positioned geometrically and treated as riding, with C—H = 0.93 Å (aromatic), 0.96 Å (methyl) and *U*_iso_(H) = 1.2U_eq_(C) and 1.5U_eq_(C) for methyl H atoms. A rotating-group model was used for the methyl.

### Crystal data

**Table:**

C_7_H_7_NO_3_	*F*(000) = 320
*M**_r_* = 153.14	*D*_x_ = 1.420 Mg m^−^^3^
Monoclinic, *P*2_1_/*c*	Mo *K*α radiation, λ = 0.71073 Å
Hall symbol: -P 2ybc	Cell parameters from 1640 reflections
*a* = 7.2429 (14) Å	θ = 3.5–27.5°
*b* = 10.347 (2) Å	µ = 0.11 mm^−^^1^
*c* = 9.898 (2) Å	*T* = 298 K
β = 105.09 (3)°	Block, colourless
*V* = 716.2 (3) Å^3^	0.30 × 0.25 × 0.20 mm
*Z* = 4	

### Data collection

**Table:**

Rigaku Mercury2 diffractometer	1640 independent reflections
Radiation source: fine-focus sealed tube	972 reflections with *I* > 2σ(*I*)
graphite	*R*_int_ = 0.053
Detector resolution: 13.6612 pixels mm^-1^	θ_max_ = 27.5°, θ_min_ = 3.5°
CCD profile fitting scans	*h* = −9→9
Absorption correction: multi-scan (*CrystalClear*; Rigaku, 2005)	*k* = −13→13
*T*_min_ = 0.96, *T*_max_ = 1.00	*l* = −12→12
7070 measured reflections	

### Refinement

**Table:**

Refinement on *F*^2^	Primary atom site location: structure-invariant direct methods
Least-squares matrix: full	Secondary atom site location: difference Fourier map
*R*[*F*^2^ > 2σ(*F*^2^)] = 0.060	Hydrogen site location: inferred from neighbouring sites
*wR*(*F*^2^) = 0.180	H-atom parameters constrained
*S* = 1.02	*w* = 1/[σ^2^(*F*_o_^2^) + (0.0868*P*)^2^ + 0.0635*P*] where *P* = (*F*_o_^2^ + 2*F*_c_^2^)/3
1640 reflections	(Δ/σ)_max_ < 0.001
100 parameters	Δρ_max_ = 0.18 e Å^−^^3^
0 restraints	Δρ_min_ = −0.16 e Å^−^^3^

### Special details

**Table:**

Geometry. All esds (except the esd in the dihedral angle between two l.s. planes) are estimated using the full covariance matrix. The cell esds are taken into account individually in the estimation of esds in distances, angles and torsion angles; correlations between esds in cell parameters are only used when they are defined by crystal symmetry. An approximate (isotropic) treatment of cell esds is used for estimating esds involving l.s. planes.
Refinement. Refinement of *F*^2^ against ALL reflections. The weighted *R*-factor wR and goodness of fit *S* are based on *F*^2^, conventional *R*-factors *R* are based on *F*, with *F* set to zero for negative *F*^2^. The threshold expression of *F*^2^ > σ(*F*^2^) is used only for calculating *R*-factors(gt) etc. and is not relevant to the choice of reflections for refinement. *R*-factors based on *F*^2^ are statistically about twice as large as those based on *F*, and *R*- factors based on ALL data will be even larger.

### Fractional atomic coordinates and isotropic or equivalent isotropic displacement parameters (Å^2^)

**Table:**

	*x*	*y*	*z*	*U*_iso_*/*U*_eq_	
N1	0.3267 (3)	0.76927 (18)	0.0785 (2)	0.0666 (5)	
C5	0.2388 (3)	1.02039 (19)	−0.0124 (2)	0.0542 (5)	
C6	0.1852 (3)	1.1517 (2)	−0.0654 (2)	0.0642 (6)	
C4	0.3492 (3)	0.99507 (19)	0.1224 (2)	0.0582 (6)	
H4A	0.3944	1.0628	0.1838	0.070*	
O3	0.3658 (3)	0.65171 (15)	0.1201 (2)	0.0955 (6)	
O1	0.2579 (2)	1.24307 (15)	0.02709 (18)	0.0782 (6)	
O2	0.0834 (3)	1.17357 (17)	−0.18032 (17)	0.0934 (7)	
C3	0.3912 (3)	0.8701 (2)	0.1644 (2)	0.0650 (6)	
H3A	0.4662	0.8542	0.2545	0.078*	
C2	0.2201 (3)	0.7926 (2)	−0.0537 (2)	0.0686 (6)	
H2A	0.1772	0.7237	−0.1140	0.082*	
C1	0.1753 (3)	0.9152 (2)	−0.0993 (2)	0.0642 (6)	
H1A	0.1010	0.9290	−0.1900	0.077*	
C7	0.2062 (4)	1.3747 (3)	−0.0153 (4)	0.1006 (9)	
H7A	0.2665	1.4325	0.0590	0.151*	
H7B	0.0699	1.3843	−0.0356	0.151*	
H7C	0.2479	1.3946	−0.0974	0.151*	

### Atomic displacement parameters (Å^2^)

**Table:**

	*U*^11^	*U*^22^	*U*^33^	*U*^12^	*U*^13^	*U*^23^
N1	0.0654 (11)	0.0577 (11)	0.0714 (12)	0.0002 (9)	0.0082 (9)	0.0014 (9)
C5	0.0466 (11)	0.0622 (13)	0.0524 (11)	0.0009 (9)	0.0105 (8)	0.0025 (9)
C6	0.0579 (13)	0.0722 (15)	0.0616 (13)	0.0056 (11)	0.0138 (10)	0.0080 (11)
C4	0.0558 (12)	0.0581 (12)	0.0557 (12)	−0.0056 (9)	0.0057 (9)	−0.0022 (9)
O3	0.1118 (15)	0.0544 (10)	0.1071 (14)	0.0042 (9)	0.0045 (11)	0.0097 (9)
O1	0.0841 (11)	0.0557 (10)	0.0839 (12)	0.0033 (8)	0.0022 (9)	0.0055 (8)
O2	0.1046 (14)	0.0930 (14)	0.0680 (11)	0.0193 (10)	−0.0035 (10)	0.0157 (9)
C3	0.0644 (13)	0.0637 (13)	0.0577 (12)	−0.0054 (10)	−0.0003 (9)	0.0027 (10)
C2	0.0688 (14)	0.0720 (15)	0.0601 (14)	−0.0032 (11)	0.0081 (11)	−0.0125 (11)
C1	0.0621 (12)	0.0753 (15)	0.0498 (11)	0.0024 (11)	0.0047 (9)	−0.0021 (10)
C7	0.108 (2)	0.0577 (15)	0.126 (2)	0.0122 (14)	0.0125 (18)	0.0178 (15)

### Geometric parameters (Å, °)

**Table:**

N1—O3	1.292 (2)	C4—H4A	0.9300
N1—C3	1.350 (3)	O1—C7	1.445 (3)
N1—C2	1.357 (3)	C3—H3A	0.9300
C5—C1	1.389 (3)	C2—C1	1.357 (3)
C5—C4	1.390 (3)	C2—H2A	0.9300
C5—C6	1.472 (3)	C1—H1A	0.9300
C6—O2	1.205 (3)	C7—H7A	0.9600
C6—O1	1.326 (3)	C7—H7B	0.9600
C4—C3	1.368 (3)	C7—H7C	0.9600
			
O3—N1—C3	121.0 (2)	N1—C3—H3A	119.1
O3—N1—C2	119.8 (2)	C4—C3—H3A	119.1
C3—N1—C2	119.1 (2)	N1—C2—C1	120.9 (2)
C1—C5—C4	117.49 (19)	N1—C2—H2A	119.5
C1—C5—C6	119.2 (2)	C1—C2—H2A	119.5
C4—C5—C6	123.3 (2)	C2—C1—C5	121.0 (2)
O2—C6—O1	123.6 (2)	C2—C1—H1A	119.5
O2—C6—C5	123.3 (2)	C5—C1—H1A	119.5
O1—C6—C5	113.05 (19)	O1—C7—H7A	109.5
C3—C4—C5	119.77 (19)	O1—C7—H7B	109.5
C3—C4—H4A	120.1	H7A—C7—H7B	109.5
C5—C4—H4A	120.1	O1—C7—H7C	109.5
C6—O1—C7	116.4 (2)	H7A—C7—H7C	109.5
N1—C3—C4	121.7 (2)	H7B—C7—H7C	109.5
			
C1—C5—C6—O2	2.1 (3)	O3—N1—C3—C4	−179.25 (19)
C4—C5—C6—O2	−177.0 (2)	C2—N1—C3—C4	1.1 (3)
C1—C5—C6—O1	−178.87 (17)	C5—C4—C3—N1	−0.6 (3)
C4—C5—C6—O1	2.0 (3)	O3—N1—C2—C1	179.22 (18)
C1—C5—C4—C3	0.1 (3)	C3—N1—C2—C1	−1.2 (3)
C6—C5—C4—C3	179.26 (17)	N1—C2—C1—C5	0.7 (3)
O2—C6—O1—C7	1.1 (3)	C4—C5—C1—C2	−0.1 (3)
C5—C6—O1—C7	−177.87 (18)	C6—C5—C1—C2	−179.32 (18)

### Hydrogen-bond geometry (Å, °)

**Table:**

*D*—H···*A*	*D*—H	H···*A*	*D*···*A*	*D*—H···*A*
C2—H2A···O2^i^	0.93	2.44	3.204 (3)	139
C4—H4A···O3^ii^	0.93	2.42	3.263 (3)	150

Symmetry codes: (i) −*x*, *y*−1/2, −*z*−1/2; (ii) −*x*+1, *y*+1/2, −*z*+1/2.
